# Proteomic Analysis of the Venom from the Ruby Ant *Myrmica rubra* and the Isolation of a Novel Insecticidal Decapeptide

**DOI:** 10.3390/insects10020042

**Published:** 2019-02-01

**Authors:** John Heep, Alica Klaus, Tobias Kessel, Maximilian Seip, Andreas Vilcinskas, Marisa Skaljac

**Affiliations:** 1 Bioresources Project Group, Fraunhofer Institute for Molecular Biology and Applied Ecology, Winchesterstrasse 2, 35394 Giessen, Germany; john.heep@ime.fraunhofer.de (J.H.); alica.klaus@mpi-bn.mpg.de (A.K.); tobias.kessel@ime.fraunhofer.de (T.K.); maximilian.seip@ime.fraunhofer.de (M.S.); andreas.vilcinskas@agrar.uni-giessen.de (A.V.); 2 Institute for Insect Biotechnology, Justus Liebig University of Giessen, Heinrich-Buff-Ring 26-32, 35392 Giessen, Germany

**Keywords:** mass spectrometry, LC-MS, Formicidae, Myrmicinae, European fire ant, antimicrobial peptide, peptide toxin, bio-insecticide, aphids, *Acyrthosiphon pisum*

## Abstract

Ants are a biodiverse group of insects that have evolved toxic venom containing many undiscovered bioactive molecules. In this study, we found that the venom of the ruby ant *Myrmica rubra* is a rich source of peptides. LC-MS analysis revealed the presence of 142 different peptides varying in molecular weight, sequence length, and hydrophobicity. One of the most abundant peaks was selected for further biochemical and functional characterization. Combined Edman degradation and de novo peptide sequencing revealed the presence of a novel decapeptide (myrmicitoxin) with the amino acid sequence NH_2_-IDPKLLESLA-CONH_2_. The decapeptide was named U-MYRTX-MRArub1 and verified against a synthetic standard. The amidated peptide was tested in a synthetic form to determine the antimicrobial activity towards the bacterial pathogens and insecticidal potential against pea aphids (*Acyrthosiphon pisum*). This peptide did not show antimicrobial activity but it significantly reduced the survival of aphids. It also increased the sensitivity of the aphids to two commonly used chemical insecticides (imidacloprid and methomyl). Since ant venom research is still in its infancy, the findings of this first study on venom peptides derived from *M. rubra* highlight these insects as an important and rich source for discovery of novel lead structures with potential application in pest control.

## 1. Introduction

There are almost 13,400 extant species of ants (Hymenoptera: Formicidae) with significant taxonomic diversity [1,2]. Many of these species are venomous [3], but little research has been carried out to determine the molecular components of ant venom due to the challenging taxonomy, the small quantity of venom available for analysis, and the widely accepted misconception that this venom is simple and contains primarily formic acid [4,5]. In contrast, venom (especially peptide toxins) from other venomous phyla, such as snakes, spiders, and scorpions, have been studied in detail [6].

Ant venom is a complex cocktail of physiologically active compounds used for both offense and defense [7]. The molecular weaponry ranges from small molecules (e.g., alkaloids), through to peptides, proteins and other substances (e.g., formic acid, biogenic amines, and salts) [8]. Given the threats posed by the antibiotic and insecticide resistance issues, there is a rising demand for novel lead structures and targets [9,10,11]. Ant venom offers a promising source for the discovery of such novel compounds, the vast majority of which remain unknown.

Most proteomic studies on ant venoms have revealed the prevalence of short linear peptides with masses below 5 kDa [3,8]. Some examples of these peptides are ponericins from the predatory ant *Pachycondyla goeldii* (reclassified as *Neoponera goeldii*) [12,13], dinoponeratoxins from the giant neotropical hunting ant *Dinoponera australis* [14] or bicarinalin from the ant *Tetramorium bicarinatum* [15]. Such compounds are frequently entitled as antimicrobial peptides (AMPs) due to their activity against a broad spectrum of pathogens, especially bacteria [12,15,16,17,18]. In addition to antimicrobial activity, these linear peptides, even if they belong to the same structural family can display different activities such as cytolytic, hemolytic, and insecticidal activity [3,8]. For example, ponericins exhibit a broad antibacterial, hemolytic, and insecticidal activity [12]. Some dinoponeratoxins have potent antibacterial activity, while few others showed no bactericidal or any other known activity [8].

There is a growing interest in venoms of insect preying animals (e.g., spiders and scorpions) as a rich source of stable proteins that could represent a new generation of insecticides that are environmentally friendly alternatives to chemical insecticides. Due to the fact that ants also use their venom to prey on insects, some of the peptide toxins have the potential to be developed as novel bio-insecticides [12,19,20]. In this regard, the linear peptide poneratoxin isolated from the venom of the ant *Paraponera clavata* has been very effective in targeting the central nervous system of insects by blocking synaptic transmission [3,21,22]. Disulfide-linked peptides are minor components in ant venom representing relatively novel structural classes of toxins with novel pharmacological properties [3,23]. Several characterized ant peptides with disulfide bonds have shown to be effective against insects, and they most commonly target ion channels [23,24]. For instance, the peptide poneritoxin Ae1a isolated from the predatory ant *Anochetus emarginatus* paralyzed sheep blowflies *(Lucilia cuprina*) [23], whereas the peptide MIITX_1_-Mg1a isolated from the giant red bull ant *Myrmecia gulosa* incapacitated cricket larvae (*Acheta domesticus*) [4].

In the past years, short linear peptides including the above mentioned AMPs and peptide toxins have attracted the attention of researchers as candidates for plant protection and pest management products [11,25,26,27]. Our previous study has shown that venom associated AMPs (originated from scorpions *Urodacus yaschenkoi* and *U. manicatus* native to Australia) greatly affected life history traits of severe agricultural pests, pea aphids (*Acyrthospihon pisum*) [28]. In addition, there is emerging evidence showing that diverse disulfide-bridged toxins isolated from venoms of spiders and scorpions can be very efficient in aphid control [29,30,31,32]. This suggests that peptide toxins could be promising components of pest management by either replacing chemical insecticides or complementing them [28].

Control of aphids still relies mainly on conventional insecticides [33]. Intensive use of these compounds has led to widespread and multiple forms of resistances in many pest insects. Some aphid species (e.g., the green peach aphid, *Myzus persicae)* are among the top resistant pests and they have developed resistance to insecticides from different chemical classes [33,34]. The emergence of resistances is also a consequence of the declining number of available active (insecticidal) compounds on the market due to regulatory changes by the European Union, the USA, and many other countries [35,36]. There were more than 1000 active compounds approved by the European Commission in 2001, whereas there were just over 250 in 2009 [35]. Therefore, the rapid occurrence of insecticide resistance among aphids together with continued restriction of available compounds calls for alternative and sustainable control measures.

The ruby ant (*Myrmica rubra*) is an aggressive, stinging ant from the subfamily *Myrmicinae* of Palearctic origin. The species is native to Europe and Asia, but more recently colonies have spread in the temperate regions of North America [37]. The species is also known as the (common) red ant, European red ant or European fire ant. However, the latter two terms are used mainly by North American researchers, where this species is considered to be adventive and a nuisance pest [38]. *M. rubra* is a scavenger and a predator that uses venom to prey on small invertebrates, including insects. It aggressively defends its territory from all invaders (e.g., mammals, birds, insects) that move through ant infested areas [39]. Prey of this ant is overwhelmed by single scouts or, if the size of the prey is too large, the nest mates will be additionally recruited following the pheromone trail of the scouts [40]. Due to the invasiveness of *M. rubra*, several studies reported a severe decline in overall insect abundance in areas of North America that are infested with this ant species [41,42,43].

Although the venom of *Myrmica* ants was already considered to be an aqueous solution of principally proteinaceous substances in 1970 [44], we are unaware of any more-recent study involving the systematic characterization of its peptide constituents. Chemical constituents such as 3-ethyl-2,5-dimethylpyrazine found in the venom gland of *M. rubra* are understood to act as pheromones or semiochemicals [44].

We investigated the venom peptidome of *M. rubra* to identify new peptides, using a combination of LC-MS and Edman degradation. Furthermore, we tested the most abundant linear peptide U-MYRTX-MRArub1 for antibacterial activity and potency against aphids as agricultural pests.

## 2. Materials and Methods

### 2.1. Ant Collection 

Ants were collected from the field (semi-open grassland) located near Schmalkalden, Thuringia, Germany. A few workers were stored in 70% ethanol for later taxonomic classification. 

In the laboratory, ants were kept in transparent plastic boxes (180 × 135 × 115 mm) containing a test tube (160 × 16 mm) with a water reservoir that served as an artificial nest. The colonies were maintained at ~23 °C and 40% relative humidity with a 16 h photoperiod. Each colony was provided twice weekly with a 20% sucrose solution and house crickets (*A. domesticus*) or mealworms (*Tenebrio molitor*) as a protein source.

### 2.2. Taxonomy

Taxonomic classification was carried out according to the identification key of Seifert [45]. Images were captured using a digital microscope (Keyence VHX-5000, Keyence, Neu-Isenburg, Germany) from various positions (head–frontal, petiolus and postpetiolus–dorsal and lateral, propodeal spine–frontodorsal).

Workers of *M. rubra* and its morphologically most similar sister species *Myrmica ruginodis* are distinguishable from all other *Myrmica* species by (i) the scapus base, (ii) the course of the scapus diameter, and (iii) the scapus length/head width ratio. There are three key characteristics to distinguish between *M. rubra* and *M. ruginodis*: (i) The shape of the petiolus, (ii) the sculpture of the postpetiolus, and (iii) the length of propodeal spines/head height ratio. From the lateral view, the petiolar node of *M. rubra* drops continuously towards the rear whereas the petiolar node in *M. ruginodis* drops in a step-like manner to form a near right angle. The sculpture of the postpetiolar node in *M. rubra* is smooth, whereas in *M. ruginodis* it is wrinkled. However, a reliable indicator to distinguish between these species is the propodeal spine length in relation to the head height, which is lower in *M. rubra*.

### 2.3. Venom Preparation

Venom gland and reservoirs of 10 foraging workers of an *M. rubra* colony were removed by dissection under a binocular microscope and were pooled in 100 µL methanol. The extracts were immediately centrifuged for 10 min at 18,000× *g* and the supernatant was transferred into a vial for crude venom fractionation or subsequent reduction followed by alkylation.

### 2.4. Disulfide Bond Reduction and Alkylation

Cysteine residues and disulfide bridges were detected after the reduction and alkylation of crude venom. Each crude venom sample was evaporated to dryness under a nitrogen stream and reconstituted in 5% acetonitrile in 50 mM ammonium bicarbonate. Dithiothreitol was added to a final concentration of 5 mM and the sample was incubated for 30 min at 56 °C to reduce disulfide bonds. The sample was cooled to room temperature. Iodoacetamide was added to a final concentration of 15 mM and the sample was placed in the dark for 30 min to alkylate the cysteines.

### 2.5. RP-HPLC Analysis of Venom Samples and Subsequent Fractionation

An appropriate sample of crude, reduced or alkylated venom was separated by reversed-phase HPLC on a DIONEX UltiMate 3000 HPLC System (Thermo Fisher Scientific, Waltham, MA, USA) with a Phenomenex Kinetex C18 analytical column (150 mm × 2.1 mm, 2.6 µm) using gradient elution with 0.1% formic acid in water (eluent A) and 0.1% formic acid in acetonitrile (eluent B). The 90 min separation was carried out at a flow rate of 0.15 mL·min^−1^ with a column temperature of 35 °C. The initial solvent mixture (5% B, 5 min) was increased to 55% B in 50 min, then to 95% B in 10 min followed by a 10 min hold, and finally a reduction to 5% B in 5 min. The column was re-equilibrated for 10 min. The UV signal was recorded using a diode array detector in the 190–450 nm range at a data collection rate of 10 Hz. The analytical run of the crude venom peptidome was divided into peak-based fractions and a suitable peak was selected for further characterization. The fractions were then lyophilized and stored at −25 °C.

### 2.6. Peptide Purification and Enrichment

Peptides were purified and enriched as previously described [46] with minor modifications. We used formic acid instead of acetic acid for buffer A (0.5% formic acid in water) and B (0.5% formic acid in water/acetonitrile 20:80). Lyophilized samples were resuspended in 100 µL of reconstitution buffer (0.1% formic acid in water/acetonitrile 95:5) and incubated for 30 min at room temperature. Briefly, C-18-StageTips were prepared by stacking two layers of Empore^TM^ C18 resin (3M) into a 200-µL pipette tip. For conditioning, 20 µL of methanol was added to the StageTip and centrifuged for 2 min at 1000× *g*. To remove contaminants, 20 µL of buffer B was added and the centrifugation step was repeated as above. The StageTip was equilibrated with 20 µL of buffer A and centrifuged as above.

Several samples representing each fraction were loaded stepwise onto the StageTip, centrifuged as above and then washed twice with buffer A. The peptides were eluted after adding 20 µL of buffer B and centrifugation as above and repeating this process. Finally, the purified and enriched peptides were lyophilized and stored at −25 °C.

### 2.7. Edman Degradation

Automated *N*-terminal sequencing was performed by stepwise Edman degradation (Proteome Factory AG, Berlin, Germany) using a Procise Model 492 cLc protein sequencer (Applied Biosystems, Foster City, CA, USA) according to the manufacturer’s protocol.

### 2.8. Peptide Synthesis

The peptide was synthesized by Romer Labs (Butzbach, Germany) according to the manufacturer’s protocol. Chloride was used as the counterion and the purity was >98%.

### 2.9. RP-HPLC of Natural and Synthetic Peptide

Natural (fractionized) and synthesized peptide were analyzed by RP-HPLC using the instrumental setup described above. The duration of the separation was reduced to 30 min. Samples were separated at a flow rate of 150 µL·min^−1^ and the column temperature was maintained at 35 °C. The initial solvent mixture (5% B, 5 min) was increased to 60% B in 16 min, then to 95% B in 3 min followed by a 3 min hold, and finally a reduction to 5% B in 1 min. The column was re-equilibrated for 5 min.

### 2.10. Analysis of Crude Venom and Fractions by Mass Spectrometry

Venom samples and fractions were analyzed using a mircrOTOF-Q II mass spectrometer (Bruker Daltonics, Billerica, MA, USA) with an electrospray ionization source in positive ionization mode. The source parameters were set as follows: End plate offset 500 V, capillary voltage 4500 V, nebulizer gas (N_2_) 1.6 bar, dry gas (N_2_) 8.0 L·min^−1^ and dry temperature 180 °C. Mass spectra were collected in the *m/z* range 250–1500.

Instrument settings for tuning were set as follows: Funnel 1 RF 200 Vpp, Funnel 2 RF 200 Vpp. isCID Energy 0.0 eV, Hexapole RF 100 Vpp, Ion Energy 4.0 eV, Low Mass 200.00 *m/z*, Collision Energy 10.0 eV, Collision RF 540 Vpp, Transfer Time 90.0 µs and Pre Pulse Storage 10.0 µs.

External calibration was performed with sodium formate clusters via the direct infusion of 10 mM sodium formate (Sigma-Aldrich, ref. 78314) prior to analysis. Additionally, a calibration segment with 10 mM sodium formate was adjusted at the beginning of each run via direct infusion (syringe pump) to perform an internal calibration. A commercial quality control standard (Waters, ref. 186006963), containing sulfadimethoxine, Val-Tyr-Val, verapamil, terfenadine, leucine-enkelphaline, and reserpine, was injected at the beginning and end of the sequence to assess the analytical parameters.

### 2.11. Antimicrobial Assay

Gram-positive (*Bacillus subtilis* DSM10, *Bacillus megaterium*, *Listeria monocytogenes* DSM20600, *Listeria fleischmanii* DSM24998, *Micrococcus luteus* DSM20030, *Staphylococcus aureus* DSM2569, and *Staphylococcus epidermidis* DSM2369) and Gram-negative bacteria (*Escherichia coli* D31 and *Pseudomonas aeruginosa* DSM50071) were cultured in lysogeny broth or tryptic soy broth as appropriate. The minimal inhibitory concentrations (MIC) were determined using a two-fold microtiter broth dilution assay in sterile 96-well plates with a final working volume of 100 µL. The cultures were incubated for 16 h at 37 °C in an ambient atmosphere. The concentration of the peptide ranged from 0.8 to 100 µM. The MIC is defined as the lowest concentration of a substance inducing complete growth inhibition and values were determined by measuring the absorbance at 600 nm. Data points were recorded every 20 min for 16 h. We tested the peptide, growth control (rifampicin) and sterility control. We have also tested the synergistic effect of U-MYRTX-MRArub1 (100 µM) with rifampicin (5 µg/mL) against *E. coli* D31 with the technical procedure as described above. All experiments were done in triplicates.

### 2.12. Maintenance of Aphids and Feeding Assays with the M. rubra Peptide

Parthenogenetic *A. pisum* (clone LL01) was maintained on the 2–3 week-old host plant *Vicia faba* var. *minor* as previously described [47,48]. Age-synchronized aphids (48 h old) were fed in modified chambers [49] for 3 days on an artificial AP3 diet [50] mixed with peptide U-MYRTX-MRArub1 (500 µg/mL) or control treatment which included the diet diluted with water. Survival was scored daily over 3 days of exposure. In total, over 2000 *A. pisum* nymphs were tested per treatment in three independent biological replicates. Aphids that survived the feeding treatments were transferred to a bioassay to investigate their susceptibility to chemical insecticides.

### 2.13. Insecticides and Aphid Bioassays

The chemical insecticides that are commonly used for aphid control were evaluated [33,51]: Imidacloprid (neonicotinoids) and methomyl (carbamates) that act on nerve and muscle targets, and spirotetramat (tetramic acid derivate) that acts on targets for lipid synthesis and growth regulation. These insecticides were purchased from Chem Service Inc. (West Chester, PA, USA). For each insecticide, a highly concentrated stock (1000 µg/mL) was prepared in acetone and afterwards, working solutions were diluted with distilled water. We have used sub-lethal concentrations determined in our previous study [52] to test the sensitivity of peptide treated aphids against each insecticide. Imidacloprid was tested at the concentration of 0.0975 µg/mL, whereas methomyl and spirotetramat were tested at a concentration of 6.25 µg/mL and 1.56 µg/mL, respectively.

Aphid bioassays were performed as previously described [52]. Briefly, bean plant stems with roots were soaked for 24 h in plastic vials containing the test treatment. Afterwards, Petri dishes with treated leaf discs were prepared as suggested by the Insecticide Resistance Action Committee (IRAC) [53]. Around 10 aphids, previously treated with peptide or diet control, were transferred onto each leaf disc in six replicates for insecticide or control treatment. Each experiment was conducted with three biological replicates. The mortality of aphids was scored after 3 days of exposure. The corresponding solvent and water controls were used during each bioassay experiment. 

### 2.14. Data Analysis

The acquired mass spectra were processed using Compass DataAnalysis v4.2 (Bruker Daltonics). A molecular feature is considered as a compound trace defined by *m/z*, retention time and intensity. A single compound may cause multiple traces but they will have both a high time-correlation and strong overlapping chromatographic peak. Molecular features were extracted from the dataset using the ‘Find Molecular Feature’ function including chromatographic peak finder v2.1 with the following parameters: S/N threshold—25, correlation coefficient threshold—0.7, minimum compound length—20 spectra, smoothing width—10.

The data related to insecticidal activities of *M. rubra* peptide or chemical insecticides were analyzed using SPSS v25 software (IBM, Armonk, NY, USA). Statistical significance was defined as *p* < 0.05. Survival data from the aphid feeding experiment were analyzed by Kaplan–Meier survival analysis and comparisons between the groups were based on log-rank tests. For the insecticide bioassays, the total mortality for each insecticide treatment was corrected according to Abbott’s formula based on mortality scored in the control (solvent) groups [54]. Mortality in the control (solvent) groups ranged between 6% and 19%. We used the Mann-Whitney U test to compare mortality between the two feeding treatments (peptide and the diet control).

## 3. Results

### 3.1. General Venom Characteristics

**Figure 1:**
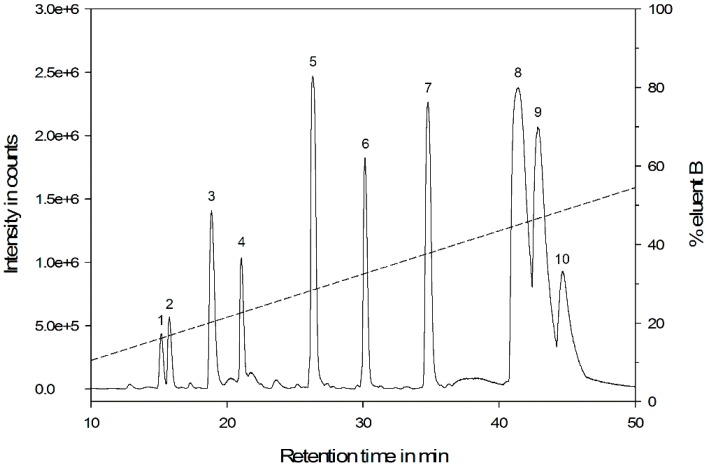
Base peak chromatogram of the venom of the ruby ant *M. rubra*. Crude venom was separated on a Kinetex C18 (150 mm × 2.1 mm, 2.6 µm, Phenomenex, USA) column using a gradient elution (dashed line) with water + 0.1% formic acid as eluent A and acetonitrile + 0.1% formic acid as eluent B. Mass spectra were recorded on a micrOTOF-QII instrument (Bruker, USA). Detailed information on numbers associated with the peaks is given in Table 1.

Crude venom was collected as a pooled sample from several *M. rubra* workers and was separated by HPLC-DAD before ESI-QTOF-MS analysis. The base peak chromatogram (Figure 1) revealed the presence of numerous compounds differing in molecular weight and hydrophobicity. Based on our acceptance criteria, we detected 233 molecular features representing 121 different compounds for individual analysis. UV absorbance at 190 and 205 nm (amide/peptide bond) indicated that the majority of the unknown substances were peptides. A large proportion of the peptides eluted between 10% and 55% acetonitrile (retention time 10–50 min), representing peptides with low to moderate hydrophobicity. The molecular weight ranged from 334 to 5348 Da, which is equivalent to peptide sequences between 3 and 49 amino acids in length. Overall, the ant venom appeared to contain a few highly abundant peptides and many more present in lower quantities. We did not observe any significant changes in molecular composition, but in some cases, the relative abundance of peptides differed among the samples coming from different *M. rubra* colonies.

### 3.2. Reduction and Alkylation

Crude venom was reduced and alkylated to obtain valuable information about the peptide structure by irreversibly severing the disulfide bonds. The reduction of a disulfide bond and the subsequent alkylation of the free cysteine residues are indicated by mass shifts of +2.0157 and +57.0220 Da, respectively. The results for the 10 major peaks (relative abundance > 10%) are shown in Table 1. *M. rubra* venom is dominated by linear cysteine-free peptides, but we also found peptides with one intramolecular disulfide bond.

### 3.3. Edman Degradation and Peptide De Novo Sequencing

The venom was divided into fractions, and we characterized in detail the peptide eluting at 26.30 min with an estimated molecular weight of 1096.6506 Da. We have chosen this peptide because this compound was free from other peptide contaminants at lower levels. The peptide was assigned using a tag based on the proposed systematic nomenclature for ant venom peptides [3] with only slight modifications. The peptide was named U-MYRTX-MRArub1 to avoid ambiguities in distinguishing peptides from species from the same or other genera (e.g., *Myrmica rugulosa* or *Manica rubida*) that would have a highly similar toxin naming. The prefix “U” is added to denote that the pharmacological target of the peptide is not known.

**Figure 2:**
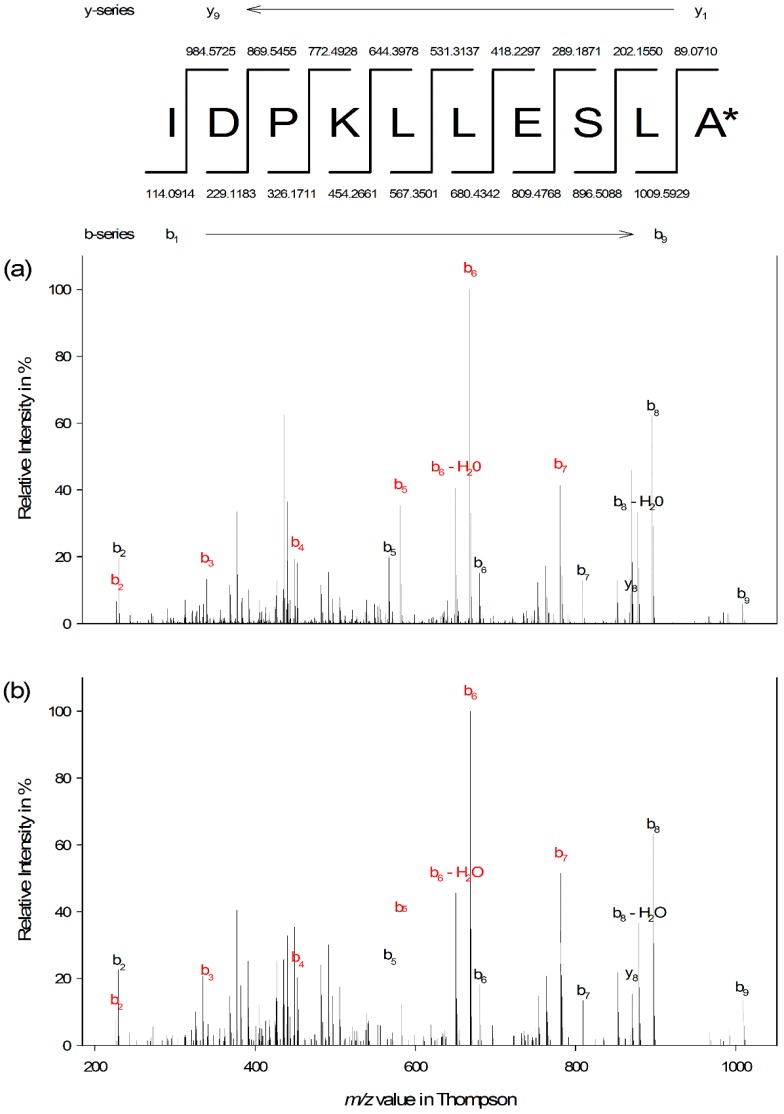
The tandem MS spectra of the natural (**a**) and synthetic (**b**) peptide U-MYRTX-MRArub1 showed highly similar fragmentation patterns. The spectrum is characterized by fragments of the native peptide (black) and the internal cleavage fragment PKLLESLA (red). Asterisk indicates C-terminal amidation. Spectra were acquired on a mircOTOF-QII instrument (Bruker, USA) by collision-induced dissociation (25 eV) with nitrogen as the collision gas.

The complete sequence of the peptide was determined by stepwise Edman degradation and verified by accurate mass measurement and MS/MS sequencing. *N*-terminal sequencing of U-MYRTX-MRArub1 yielded the decapeptide sequence IDPKLLESLA with a theoretical monoisotopic molecular weight of 1097.6333 Da. Therefore, the observed *m/z* values of 1097.6560 and 549.3326 for single- and double-charged molecular ions indicated *C*-terminal amidation, which causes mass shifts of Δ *m/z* −0.9840 and −0.4920, respectively. The sequence was confirmed by de novo sequencing of the double-charged precursor of NH_2_-IDPKLLESLA-CONH_2_ and high sequence coverage was achieved (Figure 2). The mass accuracy values for the single- and double-charged peptides were 0.62 ± 0.32 ppm and 1.04 ± 0.19 ppm (n = 3), respectively.

### 3.4. Effect of Peptide on Bacterial Strains

We did not observe any significant growth inhibition for strains tested in the antimicrobial assay. Additionally, no synergistic effect of the peptide U-MYRTX-MRArub1 with rifampicin (5 µg/mL) against *E. coli* was observed [56]. The maximum concentration of the peptide was 100 µM.

### 3.5. Effect of Peptide Treatment on A. pisum Susceptibility to Chemical Insecticides

**Figure 3:**
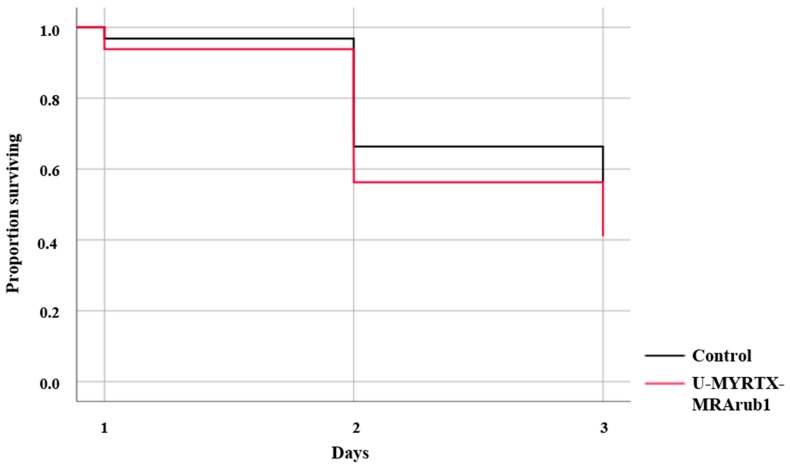
Insecticidal activity of *M. rubra* peptide U-MYRTX-MRArub1. *A. pisum* survival was monitored during 3 days of feeding on an AP3 diet mixed with the test peptide (500 µg/mL). Survival data were evaluated by Kaplan-Meier analysis and comparisons between the two groups were based on log-rank tests. Statistical data are shown in Table S1. The peptide treatment induced a small but significantly higher mortality in aphids compared to the diet control treatment (diet diluted with distilled water).

The effect of the peptide U-MYRTX-MRArub1 was first determined by tracking *A. pisum* survival during 3 days of feeding (Figure 3). Survival of aphids was significantly reduced at the tested concentration of 500 µg/mL (survival rate ≈ 40%). Aphids that survived the 3 days of peptide or control treatments were exposed to insecticide treated bean plant leaf discs in order to detect the effect of U-MYRTX-MRArub1 on aphid tolerance to three commonly used chemical insecticides, namely imidacloprid, spirotetramat, and methomyl.

**Figure 4:**
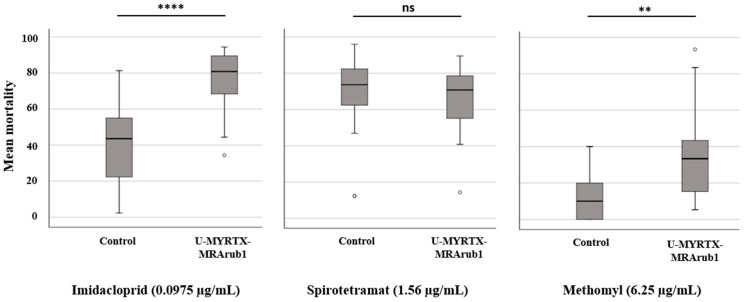
Mortality of *A. pisum*, previously exposed to feeding treatments (peptide U-MYRTX-MRArub1 and AP3 diet control), following exposure to chemical insecticides (imidacloprid, spirotetramat, and methomyl). Mortality data were evaluated by the Mann-Whitney U test. Statistical data are shown in Table S1. Statistical significance is indicated as follows: ** *p* < 0.01, **** *p* < 0.0001. ns, not significant.

Each insecticide was tested at a specific sub-lethal concentration that depended on the LC_50_ values determined in our previous study [52] (Figure 4). We found that aphids previously exposed to peptide U-MYRTX-MRArub1 were significantly more sensitive to imidacloprid (0.0975 µg/mL) and methomyl (6.25 µg/mL) compared to the diet control treatment. We observed no differences in mortality when aphids from the peptide and control treatment were exposed to spirotetramat (1.56 µg/mL) (Figure 4).

## 4. Discussion

The ruby ant *M. rubra* is a representative of the *Myrmicinae* subfamily, which comprises the greatest taxonomic diversity of stinging ants across all the subfamilies. The sting of a worker is painful and can cause local skin irritation, but as far as we know, there are no reports of fatalities due to anaphylaxis. In this study, we explored the molecular secrets of the toxins located in the venom of the ruby ant including the functional analysis of the most abundant decapeptide U-MYRTX-MRArub1.

LC-MS venom profiling of the ruby ant *M. rubra* revealed a complex mixture of peptides with variations in peptide length, molecular weight, and physicochemical properties. Overall, in the venom of *M. rubra* 10 major peptides dominated, whereas other components were present at much lower levels (Figure 1). In a triplicate analysis (Figure S1), we were able to increase the number of identified peptides from 121 to 142 (+17%, for a complete mass list, see Supplementary Materials Table S2). Although some of the peptides were identified only once (50 from 142; 35%), most were detected repeatedly (92 from 142, 65%) resulting in a good peptidome coverage. These findings underline the necessity of highly sensitive and accurate MS instrumentation for the in-depth analysis of ant venom peptides. Some of the low abundant components may be contaminants from the tissues surrounding the venom gland and reservoirs. However, the complexity of our data agrees with previous studies showing, for instance, the peptide richness of the venom of the ants *Dinoponera australis* [14] and *Dinoponera quadriceps* [57].

Triplicate single-run analysis of crude venom also revealed a heterogeneous distribution of molecular weights ranging from 334 to 5348 Da (Figure S2a). Interestingly, we only found a few peptides in the higher (>3500 Da, 1 peptide, 0.7%) and lower (<1000 Da, 19 peptides, 13.4%) molecular weight areas, whereas the majority of *M. rubra* venom peptide toxins were between 1000 and 3500 Da (122 peptides, 85.9%). These results are consistent with those of Touchard et al. [58] who found that many ant venoms contain small peptides with a molecular weight of 500–4000 Da. The results of disulfide mapping also agree with the abovementioned study. We monitored the presence of disulfide bonds for the 10 major peptides, and disulfide-linked peptides appeared to be less substantial because only two peptides contained one intramolecular disulfide bond. The structural diversity of peptide toxins seemed to be restricted to linear and cysteine-free peptides. The mean and median putative peptide toxin in the venom of *M. rubra* has a molecular weight of 1819 and 1602 Da, which corresponds to peptide sequence lengths of 16 and 14 amino acids, respectively. The peptide length was estimated using the molecular weight of averagine (111.1254 Da), a theoretical average amino acid sequence based on the statistical abundance of each amino acid [55]. As shown in Figure S2b, the estimated peptide length for peptides within the interquartile range is in a notably narrow window, from 10 to 24 amino acids. The majority of the putative toxins (1000–3500 Da) are peptides between 9 and 31 amino acids in length, revealing a similarity to peptide toxins from cone snails (generally between 10 and 30 amino acids) but we did not observe the exceptionally high number of disulfide bonds as found in conotoxins [59]. In contrast, the venom toxins of other venomous phyla such as snakes, spiders, or scorpions are typically between 40 and 100 amino acids in length [60].

Ant venom research is still in its infancy, and the nomenclature system for novel peptide toxins is therefore inconsistent. In the future, the use of highly sophisticated analytical instrumentation and integrated omics technologies will increase the number of newly discovered peptide toxins. The absolute number of venom-derived peptide toxins is relatively low compared to those described in other venomous phyla, such as snakes and scorpions [10,61]. However, peptide toxins have been named according to various characteristics (e.g., biological source or similar functions) resulting in multiple names for the same peptide. Therefore, we highly recommend the introduction of a systematic nomenclature system for peptide toxins as proposed by Touchard et al. [3]. This system includes information about biological properties, evolutionary and taxonomic characteristics, and the number of identified peptides. The overriding objective of such a nomenclature system is to condense information and generate an unambiguous identifier for peptide toxins. Within the taxonomically diverse *Myrmicinae* subfamily, there are inevitably many taxa with similar names at both the genus and the species level, and these would necessitate a long and inconvenient peptide toxin terminology. Therefore we have introduced the abbreviation “MRA” for the *Myrmica* genus and “rub” for the *M. rubra* species. This will avoid confusion with other genera with similar names (e.g., *Myrmecina*, *Myrmicaria,* and *Myrmicocrypta*) and with similarly-named species within the *Myrmica* genus (e.g., *M. ruginodis*) to generate a concise peptide toxin terminology. As previously stated, the prefix “U” indicates that pharmacological target is unknown.

We isolated and characterized a novel peptide from the venom of *M. rubra* workers. U-MYRTX-MRArub1 is a linear, cysteine-free decapeptide and the C-terminus is post-translationally modified by amidation. C-terminal amidation is a widespread post-translational modification of AMPs that has been observed in ants [8] and many other eukaryotic organisms [62,63,64,65,66]. This modification may enhance peptide stability and antimicrobial activity [67]. The share of hydrophobic residues is 60% and the overall net charge is ± 0. The presence of acidic (D, E) and basic (K) amino acids creates both amphoteric and amphipathic properties.

Most AMPs that have been described thus far are cationic and are considered as promising leads for the development of novel antibiotics and therapeutics, and also for other applications such as bio-insecticides [28,68,69]. However, here we present a novel peptide with a neutral net charge.

We performed a homology search using protein BLAST in ExPASy and the Antimicrobial Peptide Database [70]. Some parts of the sequence matched bacterial enzymes such as peptidases or kinases (ExPASy). The results for Antimicrobial Peptide Database homology search revealed a high sequence homology to vespid chemotactic peptides and temporins (Figure S3). Vespid chemotactic peptides have been recently isolated from the venom gland of the social wasp *Vespa tropica* [71]. The temporins are a family of AMPs originally isolated from the skin secretions from the European common frog *Rana temporaria* [72]. Our myrmicitoxin shares 50% sequence identity and 70% sequence similarity with VCP-VT1 and the temporins B, D, E, H, and K. Interestingly, the peptide U-MYRTX-MRArub1 shares both the same sequence length of 10 amino acids and the C-terminal amidation with the abovementioned peptides. ^6^Leu is ubiquitous to all AMPs and, interestingly, the amino acids ^5,6^Leu, ^8^Ser and ^9^Leu share a common motif with the temporin family. A recent study on the venom of the myrmicinae ant *T. bicarinatum* has revealed the presence of numerous peptides [5]. The newly discovered peptide U_12_-MYRTX-Tb1a shares 50% sequence identity, 70% sequence similarity, sequence length and C-terminal amidation with our myrmicitoxin U-MYRTX-MRArub1. However, these peptides share a different motif: ^3^Pro, ^6^Leu, ^8^Ser, ^9^Leu, ^10^Ala (Figure S3). Surprisingly, the abundance of U_12_-MYRTX-Tb1a in the mass spectrometry profile of the crude venom was only of low relative abundance (~1.7%), whereas U-MYRTX-MRArub1 from this study was the most abundant peptide in the venom profile of *M. rubra*.

We performed a structural prediction using the Peptide Structure Prediction Server (PEP-FOLD 3) [73]. The generated model of U-MYRTX-MRArub1 displayed a helical conformation of the peptide. The helical wheel projection of U-MYRTX-MRArub1 and U_12_-MYRTX-Tb1a (Figure S4) is promoting the structural similarity between these peptides. For both peptides, hydrophobic amino acids are predominant on one side of the predicted projection, whereas acidic or hydroxylic amino acids are primarily located on the other side of the helix, underlining its amphipathic character. This property is necessary for α-helical membrane-interacting AMPs to insert a hydrophobic section into the lipid bilayer, allowing them to form pores and cause bacterial cell lysis [74].

The antimicrobial activity of the chemically synthesized peptide was tested against a broad range of bacteria. We did not observe any significant growth inhibition of *B. megaterium*, *B. subtilis*, *L. fleischmanii*, *L. monocytogenes*, *M. luteus*, *S. aureus*, *S. epidermidis*, *E. coli* or *P. aeruginosa* at concentrations up to 100 µM. The absence of antimicrobial activity in U-MYRTX-MRArub1 supports evidence from previous research in which the structurally similar temporin H was found to be inactive against bacterial strains [72]. Temporin H was shown to alter the permeability of bacterial inner and outer membranes, but it was unable to lyse the bacteria. In addition, the antimicrobial activity of rifampicin against *E. coli* D21 was 10-fold higher in the presence of temporin H than without [75], whereas this synergistic effect was not observed in our study. In agreement with our findings, few temporin-like peptides isolated from the giant ant *Dinoponera quadriceps* were either inactive against various bacteria, fungi, and yeasts or had only minor activity against some of the evaluated bacteria [57]. On the other side, ponericins, peptides isolated from the venom of the predatory ant *P. goeldii* showed activity against Gram-positive and Gram-negative bacteria, including *S. aureus*, *E. coli* and others [12]. These peptides are structurally different from U-MYRTX-MRArub1 and we could not find any homology. In order to fully understand the antimicrobial activity of U-MYRTX-MRArub1, additional studies will be needed to define the peptide’s activity on bacterial membranes. It may also be that U-MYRTX-MRArub1 is active against some other microorganisms that were not tested in this study.

In this study, we have tested the potential of the synthetic peptide U-MYRTX-MRArub1 against the well-known model insect and a pest organism, *A. pisum.* We have observed the insecticidal activity of the tested peptide after its oral delivery (Figure 3). However, we assume that the effective concentration of the ant peptide will be even lower if the synthetic peptide is injected. The mortality of the peptide treated aphids in this study was higher after exposure to sub-lethal concentrations of imidacloprid and methomyl than in the control group which was not previously exposed to U-MYRTX-MRArub1 (Figure 4).

Current literature is not reporting any known cytotoxic or hemolytic activity of temporin H that is structurally similar to U-MYRTX-MRArub1. We can assume that these two peptides have similar biological activity following their structural similarities. Linear peptides including AMPs are known to act on different targets (e.g., cell division, macromolecular synthesis, gene translation) and not only membranes [76]. The natural characteristics of the U-MYRTX-MRArub1 suggest that its insecticidal function was probably not a result of membrane disruption, but rather an activity on some other molecular target. 

Venomous animals frequently contain neurotoxic peptides assisting in immobilization of the prey. Such peptides act on diverse biological targets, mainly ion channels [3]. Poneratoxin originated from the ant *P. clavata* is capable of modulating voltage-gated sodium (Na_V_) channels of both vertebrates and invertebrates [3,20]. Another structurally more complex neurotoxic peptide, Ectatomin Et-1 from the ant *Ectatomma tuberculatum*, is a voltage-gated calcium (Ca_V_) channel blocker and it acts as a pore-forming peptide on eukaryotic cells [3]. 

Follow-up studies should investigate whether U-MYRTX-MRArub1 acts of ion channels or some other biological targets to get the complete picture on functional activities, including the one against aphids. Sometimes highly active venom originated peptides can be used as stand-alone bio-insecticides, but more frequently they may be more efficiently delivered to the target pest through engineered insect-resistant crops or transgenic entomopathogens (e.g., fungi, baculoviruses) with enhanced activity [10]. Application strategy depends on the potency of the peptide against target pests, but also its stability towards degradation of host proteases. Therefore, peptides are frequently not applied in their natural structure, but their features are additionally improved by amino acid alterations [11,77]. Short and linear peptide molecules such as U-MYRTX-MRArub1 are especially attractive candidates for the development of novel bio-insecticides because they are easy to synthesize at low costs [28].

## 5. Conclusions

This work significantly contributes to the largely unstudied field of ant venom research. Herein, we present an extensive analysis of peptides in the venom of the myrmicine ant *M. rubra* and functional characterization of the most abundant peptide U-MYRTX-MRArub1. Further studies will reveal more detailed characterization of the other putative peptide toxins present in the venom of *M. rubra* and clarify whether it is required for some of the peptides to act synergistically to produce additional biological effects. A newly discovered decapeptide U-MYRTX-MRArub1 impaired fitness of major agricultural pest insects and therefore offers new perspectives in integrated pest management.

## Figures and Tables

**Table 1:** Major peptides with a relative abundance >10% in the venom of the ruby ant *M. rubra*. Most of the peptides are linear, but reduction and alkylation of crude venom revealed the presence of two peptides with one intramolecular disulfide bond (highlighted in gray). RT: Retention time, MW: Molecular weight, RA: Relative abundance, aa: Amino acid, S-S: Disulfide bridges.

No.	RT (min)	MW_crude_ (Da)	MW_red._ (Da)	MW_alk._ (Da)	RA (%)	aa Length ^a^	S-S
1	15.17	544.2524	544.2518	544.2525	18	5	0
2	15.77	1100.6561	1100.6547	1100.6544	23	10	0
3	18.84	1463.8353	1465.8501	1579.8809	57	13	1
4	21.05	1401.7441	1401.7418	1401.7336	42	13	0
5	26.30	1096.6506	1096.6470	1096.6487	100	10	0
6	30.15	1112.6765	1112.6713	1112.6750	74	10	0
7	34.78	1590.8308	1592.8418	1706.8684	92	14	1
8	41.41	2477.4727	2477.4578	2477.4604	41	23	0
9	42.83	2837.5846	2837.5700	2837.5726	84	26	0
10	44.66	2525.4244	2525.4101	2525.4146	38	23	0

^a^ peptide length was determined using averagine (111.1254 Da) [55].

## Supplementary Materials

The following are available online at http://www.mdpi.com/2075-4450/10/2/42/s1, Table S1: Summary of statistical data measured for insecticidal activity in this study, Table S2: Peptide mass list from crude venom analysis of *M. rubra*, Figure S1: Technical replicate of a crude venom sample of the ruby ant *M. rubra*, Figure S2: Box-and-whisker plot of (a) molecular weight and (b) peptide sequence length distribution, Figure S3: Sequence alignment of U-MYRTX-MRArub1 with other antimicrobial peptides/peptide toxins, Figure S4: Helical wheel projection of U-MYRTX-MRArub1 and its most closely related ant-derived peptide homolog, U_12_-MYRTX-Tb1a.
