# Supplementary material for: Proteomic Analysis of the Venom from the Ruby Ant Myrmica rubra and the Isolation of a Novel Insecticidal Decapeptide
Source: Insects. 2019 Feb 1;10(2):42. doi: 10.3390/insects10020042 (PMC6409562; doi:10.3390/insects10020042)
Supplement: Supplementary file 1 [file insects-10-00042-s001.pdf]

insects

Supplementary

Proteomic Analysis of the Venom from the Ruby Ant *Myrmica rubra* and the Isolation of a Novel Insecticidal Decapeptide

John Heep [1], Alica Klaus [1], Tobias Kessel [1], Maximilian Seip [1], Andreas Vilcinskas [1,2] and Marisa Skaljac [1,*]

[1] Bioresources Project Group, Fraunhofer Institute for Molecular Biology and Applied Ecology, Winchesterstrasse 2, 35394 Giessen, Germany; john.heep@ime.fraunhofer.de (J.H.); alica.klaus@mpi-bn.mpg.de (A.K.); tobias.kessel@ime.fraunhofer.de (T.K.); maximilian.seip@ime.fraunhofer.de (M.S.); andreas.vilcinskas@agrar.uni-giessen.de (A.V.)

[2] Institute for Insect Biotechnology, Justus Liebig University of Giessen, Heinrich-Buff-Ring 26-32, 35392 Giessen, Germany

* Correspondence: marisa.skaljac@ime.fraunhofer.de

Received: 08 January 2019; Accepted: 24 January 2019; Published: date

Table S1. Summary of statistical data measured for insecticidal activity in this study.

**Treatment**	**Survival during the 3 days feeding on an AP3 diet (control) alone or mixed with the peptide (see Figure 3)**				**Significance**
	**Mean**				
	**Estimate**	**Standard error**	**95% Confidence Interval**		
			**Lower Bound**	**Upper Bound**	
**Control**	4.63	0.01	4.61	4.66	Control vs. U-MYRTX-MRArub1 $p < 0.0001$
U-MYRTX-MRArub1 (500 µg/mL)	4.50	0.01	4.48	4.52	

**Chemical insecticides (concentration)**	**Treatment/mortality§ after exposure to chemical insecticides (see Figure 4)**		**Significance**
	Control	U-MYRTX-MRArub1	
Imidacloprid (0.0975 µg/mL)	39.55±5.10	76.35±4.30	$p < 0.0001$
Spirotetramat (1.56 µg/mL)	67.75±4.58	66.14±4.81	ns
Methomyl (6.25 µg/mL)	11.49±3.09	35.69±6.60	$p < 0.01$

§ Mean±SE, ns—not significant.

Table S2. Peptide mass list from crude venom analysis of *M. rubra*. In total, 142 different peptides were found in a triplicate analysis. Masses are displayed as molecular weights (Da). Mass in bold was subjected to further characterization.

Molecular weight in Da					
334.1769	338.1709	354.0727	354.2384	445.1841	476.7588
494.2727	526.242	528.2567	544.2525	616.3656	616.5762
621.1135	796.4795	869.5237	970.5428	980.5902	987.5693
990.5738	1006.6039	1008.5845	1024.6151	1026.5945	1042.6256
1048.5759	1055.6356	1079.6213	1083.6311	1095.653	**1096.6509**
1097.2811	1098.2848	1100.657	1101.6396	1112.6777	1112.6819
1113.3002	1119.6201	1134.6615	1135.5856	1150.5540	1150.6291
1170.6559	1180.6439	1180.6459	1182.6479	1237.6649	1249.6347
1269.7229	1279.7034	1281.7579	1284.7638	1285.6493	1297.7186
1324.773	1337.7813	1350.7481	1364.6612	1366.7013	1384.7179
1401.7451	1402.7269	1422.8039	1423.7251	1439.6918	1455.6609
1463.8374	1477.7462	1521.9032	1556.8036	1590.8348	1612.8095
1628.7758	1635.9156	1645.9045	1663.9113	1682.9534	1703.8584
1718.8845	1762.9111	1774.9512	1776.8915	1790.9099	1855.0609
2097.2195	2106.3675	2106.3687	2166.995	2232.1919	2263.3008
2307.3526	2318.4453	2355.3197	2477.4680	2493.4531	2499.4415
2515.4105	2521.4224	2525.425	2535.4619	2537.4662	2538.4287
2541.4185	2547.4066	2563.3787	2569.3936	2639.5092	2650.5502
2677.5528	2703.5555	2726.3719	2751.5208	2753.7302	2815.2691
2823.6714	2837.5828	2859.5571	2865.7696	2866.5074	2866.8204
2867.8117	2875.5257	2881.5363	2883.7975	2884.8193	2884.8208
2890.4966	2892.5284	2907.5849	2936.5159	2999.6460	3000.6428
3037.6609	3055.6859	3064.5863	3079.8785	3080.9258	3082.585
3096.8974	3298.6299	3314.7518	5348.2571		

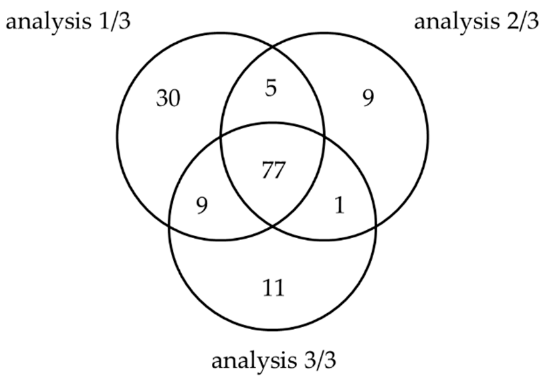

Figure S1. Number of identified peptides in three technical replicates of a crude venom sample of the ruby ant *M. rubra*.

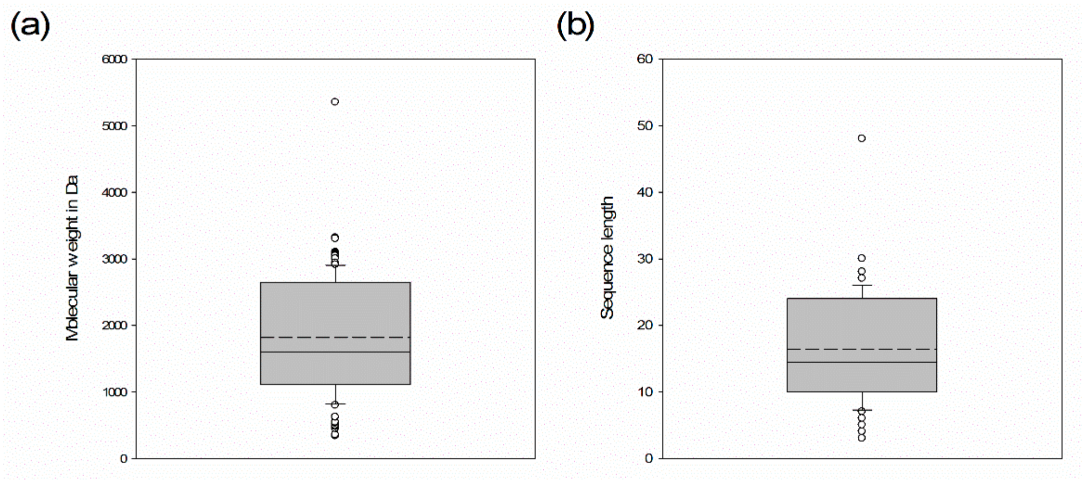

Figure S2. Box-and-whisker plot of **(a)** molecular weight and **(b)** peptide sequence length distribution. Sequence length is obtained by dividing the molecular weight of a peptide by the mass of averagine (111.1254 Da), a modeled average amino acid based on the natural occurrence of each amino acid [1]. Boundaries of the box show the 25th and 75th percentiles, whiskers delineate the 10th and 90th percentiles, median and mean are outlined as solid and dashed lines, respectively.

Organism	Name				1				5					10		aa length	S%	I%
Myrmica rubra	U-MYRTX-MRArub1	-	-	-	I	D	P	K	L	L	E	S	L	A	*	10	100	100
Tetramorium bicarinatum	U_{12}-MYRTX-Tb1a	-	-	-	L	S	P	A	V	L	A	S	L	A	*	10	70	50

Organism	UniProtKB	APD ID	Name				1				5					10		aa length	S%	I%
Myrmica rubra			U-MYRTX-MRArub1	-	-	-	I	D	P	K	L	L	E	S	L	A	*	10	100	100
Rana temporaria	P79876	AP00859	Temporin-H	-	-	-	L	S	P	N	L	L	K	S	L	L	*	10	70	50
Rana temporaria	P56923	AP00100	Temporin-K	-	-	-	L	L	P	N	L	L	K	S	L	L	*	10	70	50
Rana temporaria	P56920	AP00097	Temporin-E	V	L	P	I	I	G	N	L	L	N	S	L	L	*	13	70	50
Rana temporaria	P79874	AP00095	Temporin-B	L	L	P	I	V	G	N	L	L	K	S	L	L	*	13	70	50
Rana temporaria	P56919	AP00858	Temporin-D	L	L	P	I	V	G	N	L	L	N	S	L	L	*	13	70	50
Vespa tropica		AP02368	VCP-VT1	F	L	P	I	I	G	K	L	L	S	G	L	L		13	70	50
Hylarana taipehensis		AP02467	Temporin-LF1	F	L	P	F	V	G	K	L	L	S	G	L	L		13	70	40
Rana temporaria	P56921	AP00098	Temporin-F	F	L	P	L	I	G	K	V	L	S	G	I	L	*	13	70	20
Rana temporaria	P79875	AP00099	Temporin-G	F	F	P	V	I	G	R	I	L	N	G	I	L	*	13	70	10
Rana temporaria	P56917	AP00094	Temporin-A	F	L	P	L	I	G	R	V	L	S	G	I	L	*	13	70	10
Rana pirica		AP01259	Temporin-1PRb	I	L	P	I	L	G	N	L	L	N	S	L	L	*	13	70	50
Rana temporaria	P56918	AP00096	Temporin-C	L	L	P	I	L	G	N	L	L	N	G	L	L	*	13	60	40
Vespa tropica		AP02369	VCP-VT2	F	L	P	I	I	G	K	L	L	S	G	-	-		11	50	40
Pelophylax saharica		AP00899	Temporin-SHb	F	L	P	I	V	T	N	L	L	S	G	L	L	*	13	50	40

Figure S3. Sequence alignment of U-MYRTX-MRArub1 with other antimicrobial peptides/peptide toxins. Identical amino acids are highlighted in green boxes and conservative substitutions in blue boxes. Sequence similarity (S%, with conservative substitutions) and sequence identity (I%, without conservative substitutions) are relative to MYRTX-MRArub1. Parameters for conservative

substitutions: Positive (R, K,), negative (D, E), hydrophobic (V, I, L, A, F, P, W, G). The red frame is used to emphasize a common motif within structural related peptides. Asterisks indicate C-terminal amidation.

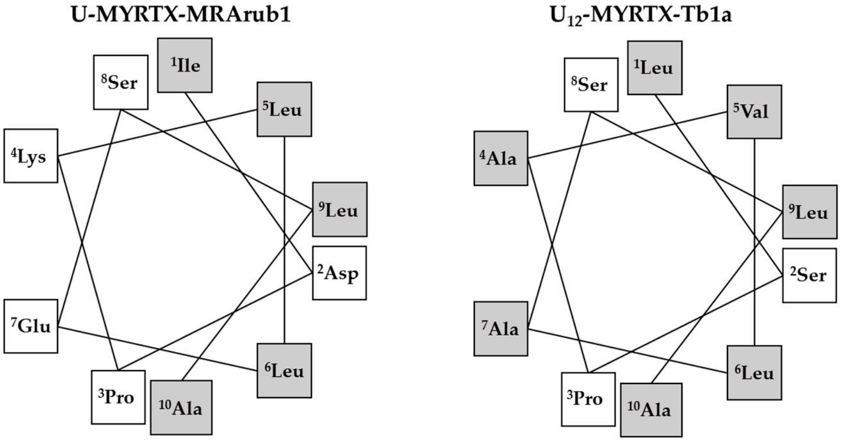

Figure S4. Helical wheel projection of U-MYRTX-MRArub1 and its most closely related ant-derived peptide homolog, U_{12}-MYRTX-Tb1a [2]. Hydrophobic amino acid residues are highlighted in gray.

References

1. Senko, M.W.; Beu, S.C.; McLafferty, F.W. Determination of monoisotopic masses and ion populations for large biomolecules from resolved isotopic distributions. *J. Am. Soc. Mass. Spectrom.* **1995**, *6*, 229–233. doi:10.1016/1044-0305(95)00017-8.
2. Touchard, A.; Téné, N.; Song, P.C.T.; Lefranc, B.; Leprince, J.; Treilhou, M.; Bonnafé, E. Deciphering the Molecular Diversity of an Ant Venom Peptidome through a Venomics Approach. *J. Proteome Res.* **2018**, *17*, 3503–3516. doi:10.1021/acs.jproteome.8b00452.

© 2019 by the authors. Submitted for possible open access publication under the terms and conditions of the Creative Commons Attribution (CC BY) license (http://creativecommons.org/licenses/by/4.0/).
